# Ninth Biennial Report of the Officers of the Illinois State Hospital for the Insane

**Published:** 1865-04

**Authors:** 


NINTH BIENNIAL REPORT OF THE OFFICERS OF THE ILLINOIS STATE HOSPITAL FOR THE INSANE.

We have just received this official document, in regard to one of our most important institutions. It affords evidence that the State Hospital for the Insane has been well managed in all its departments. In a hasty perusal of the Superintendent's report, we find many facts and observations of interest and importance. The following table will show the number of patients treated in the institution, and the results, during the last two years:—

| | |
|---|---|
| Number of patients in the hospital Dec. 1, 1862 | 302 |
| Number since admitted | 408 |
| Whole number treated since Dec. 1, 1862 | 710 |
| Discharged, recovered | 159 |
| Discharged, unrecovered, by order of Trustees | 133 |
| Discharged, unrecovered, by mutual consent of Superintendent and committing parties | 48 |
| Discharged, unrecovered, but improved | 14 |
| Eloped | 13 |
| Died | 42 |
| Total vacancies created | 409 |
| Remaining Dec 1, 1864 | 301 |

On the important subject of improper commitments, and unjust detentions of patients in asylums, he makes the following quotations from the report of a Commission of Lunacy in Massachusetts. They show that the suspicions often entertained by some in the community on this subject, have little or no foundation, in fact:—

In allusion to the whole question of wrongful commitments, the language of the commissioners is as follows:—

"With reference to the above points, the commissioners would frankly acknowledge that no such case of clearly wrongful confinement, in a hospital, has been brought to their notice, and but a single instance of wrongful admission. This was at the instance of a Judge of Probate, and was discharged at once by the superintendent, upon learning the facts of the case."

In regard to the question, "Shall the internal management of hospitals be regulated by special statute?" the committee lays down the following dicta:—

"The interior management of hospitals, and the treatment of the insane, cannot be regulated by law. It would be as absurd and futile to attempt, by statute, to regulate the minute and subtle details of mental hygiene and therapeutics in our hospitals, as it would be to legislate how physicians should treat fever, or how, or when, a surgeon should amputate a case of gangrene, or, even, to place on the statute book laws, with penalties, for guiding the practice of a shipmaster when in peril of shipwreck, with hundreds of alarmed passengers dependent for safety on his free will, cool head, and skilful hand. The entire management and treatment of the insane must be confided to the humanity and skill of the superintendent. His authority must be personal. There can be no divided responsibility in the medical treatment of the insane."

The following conclusions of the committee are full of value, and corroborate the views of those most conversant with good and bad influences upon the insane.

"A number of cases have been brought to the notice of the commission, where the unadvised visits of friends have recalled feelings and associations of which time and change of circumstances had effected the removal, and when a continuance of the same system might, if uninterfered with, have led to a permanent cure. The first condition of restoration is, that the patient be separated from all the scenes, ideas, and associations amid which, or out of which, his insanity arose, and which tend to keep up his delusions, excitements, or depressions. The faithful physician avoids even allusion to these. He discourages conversation upon them, and yet the patient's proclivity is towards them, and he will talk about them if he can find any one to listen to him. But he prefers to write to his friends, for he can talk to them though absent. His letters, therefore, excite him, and keep him in a morbid state of mind. Under such circumstances, the trustworthy manager of the insane discourages the practice, in the early stages of treatment, and until he sees it can be done without detriment to the patient's health."

On the very important subject of giving testimony in courts, as a medical expert, he introduces the following very important suggestion, which *ought* to be acted upon by the State Legislatures, without unnecessary delay:—

The author just quoted, has in a spirit of returning candor, very clearly stated the very remedy which those who suffer, in rendering testimony under the present state of things have long desired.

"There must be," he says, "something fatally defective in our mode of obtaining and applying this class of testimony. For it cannot be supposed that, under proper regulations, there could be any difficulty in obtaining reliable scientific evidence, if the proper methods were resorted to. And it seems to us that some mode should be devised whereby the motive which is now offered to this class of witnesses to testify so exclusively for one side, should be not only counteracted, but that it should be entirely removed and a contrary motive, for impartiality, presented. We mean no impeachment of this class of witnesses; but any man when approached by the counsel of one party, and furnished only with the views and facts of one side, and asked to give his opinion, naturally gives a one-sided opinion. And, having committed himself to one side, he is thereafter rendered incapable of forming a fair and unbiased judgment upon the facts. He becomes disqualified to act as a juror in the case. And when it is considered that his testimony is given to instruct, educate, and inform the court and jury, in regard to the proper mode of determining the case, and that it is no uncommon occurrence for a case to turn very much upon the scientific and professional testimony, it is no less important that the experts should be wholly uncommitted in opinion, than that the jurors should be so. It seems very obvious, therefore, that this class of witnesses should be selected by the court, and that this should be done wholly independent of any nomination, recommendation, or interference of the parties, as much so, to all intents and purposes, as are the jurors. To this end, therefore, should the compensation of scientific experts be fixed by statute, or by the court, and paid out of the public treasury, and either charged to the expense of the trial, or part of the costs of the cause, or not, as the Legislature should deem the wisest policy. The mere expense of the experts, when selected in this mode, would be as nothing in comparison with the expense which now becomes unavoidable, in consequence of the enormous consumption of time in most of the trials of this class."

The undersigned would respectfully urge, in view of the unquestionable propriety of this measure of reform, that the Legislature be requested to amend sec. 16 of the act of Feb. 15, 1851, (which, in part, establishes the relation of the Superintendent of this institution to the courts of the State,) in the manner above indicated; or, else, to make complete the power, now in part existing, of being beyond the reach of any subpœna which would impress his services as an *expert.*

---

Sour Milk to Irritable Ulcers.—Dr. Bélin, of l'Assomption, tells us that he has been in the habit, for many years, of treating old inflamed ulcers, and those with exuberant granulations, by means of cold poultices of coagulated milk kept constantly to the part, and renewed several times a day. He says that he has often found them speedily to reduce inflammation, and heal ulcers that have resisted every other mode of treatment for months.

